# Characterization of pathogens and prognostic factors in cerebral infarction patients complicated by pulmonary infections

**DOI:** 10.1097/MD.0000000000045309

**Published:** 2025-11-14

**Authors:** Suxia Hu, Zejin Wang, Lei Zhu, Yifei Zhu

**Affiliations:** a Department of Clinical Laboratory, The First Hospital of Anhui University of Science and Technology, Huainan, Anhui, People’s Republic of China; b School of Medicine, Anhui University of Science and Technology, Huainan, Anhui, People’s Republic of China; c Department of Neurology, The First Hospital of Anhui University of Science and Technology, Huainan, Anhui, People’s Republic of China.

**Keywords:** cerebral infarction, logistic regression, nomogram, pathogenetic features, prognostic factors, pulmonary infection

## Abstract

This study aimed to investigate the microbial spectrum and prognostic indicators in patients who developed pulmonary infections secondary to cerebral infarction (CI). A total of 175 retrospective cases with post-CI pneumonia were reviewed. The study was conducted at the First Affiliated Hospital of Anhui University of Science and Technology, a single tertiary care center, between October 2023 and October 2024. Pathogenic organisms were identified, and patients were stratified into favorable (n = 116) and unfavorable (n = 59) outcome groups based on clinical prognosis. Variables with significant differences in univariate analysis were further assessed using multivariate logistic regression. A prognostic nomogram was constructed, and its predictive efficacy was evaluated using the area under the receiver operating characteristic curve (AUC). A total of 169 bacterial strains were isolated, with gram-negative organisms comprising 63.31% (107/169). Twenty-eight cases involved polymicrobial infections. *Acinetobacter baumannii* demonstrated high antimicrobial resistance, showing 95.5% resistance to carbapenems, 90.9% to cephalosporins, and 86.4% to aminoglycosides. Statistically significant differences between the prognosis groups were observed in ICU admission status, presence of atrial fibrillation, consciousness level, invasive interventions, neutrophil-to-lymphocyte ratio (NLR), C-reactive protein/albumin ratio (CAR), and occurrence of multiple infections (*P* < .05). Logistic regression confirmed atrial fibrillation, polymicrobial infections, invasive procedures, elevated NLR, and higher CAR as independent predictors of poor clinical outcome (*P* < .05). The nomogram exhibited strong predictive capability with an AUC of 0.883 (95% CI: 0.828–0.937), sensitivity of 83.1%, and specificity of 86.2%. Internal validation via 1000 bootstrap replicates yielded a comparable AUC of 0.881 (95% CI: 0.830–0.931). Gram-negative bacteria are the predominant pathogens in pulmonary infections following CI, and coinfections are relatively frequent. Several clinical and inflammatory markers (particularly atrial fibrillation, polymicrobial infection, invasive procedures, NLR, and CAR) serve as independent prognostic factors. Early recognition and intervention targeting these parameters may improve clinical outcomes in this high-risk patient population. In clinical practice, serial monitoring of NLR and CAR (e.g., every 48 hours) may help detect early deterioration and guide timely interventions, such as ICU transfer, adjustment of antimicrobial regimens, and intensified supportive care.

## 1. Introduction

Cerebral infarction (CI) is a prevalent cerebrovascular disease that presents a significant challenge in clinical practice due to its high lethality and long-term disability. Epidemiological data indicate a rising incidence of CI globally, which underscores the increasing burden on healthcare systems and society at large.^[1,2]^ The primary mechanism underlying CI is the obstruction of cerebral blood vessels, leading to an interruption in the cerebral blood supply. This ischemic event results in reduced oxygen delivery, thereby inducing a cascade of pathophysiological processes including ischemia, hypoxia, and eventual necrosis of brain tissue. In the context of global mortality, CI ranks as the second leading cause of death following ischemic heart disease, further emphasizing its clinical and public health importance.^[3]^

Pulmonary infection (PI) frequently occurs as a complication in patients who have experienced a CI. Clinical studies have identified PI as a major contributor to adverse outcomes in this patient population, with infection rates reported between 7% and 38% within the first week following CI.^[4]^ The early onset of PI, typically developing within 2 to 7 days after the cerebral event, exacerbates the clinical course by further impairing patient recovery and increasing mortality. The coexistence of CI and PI presents a complex interplay between neurological and respiratory pathologies, where the systemic inflammatory response and immune dysregulation may potentiate the severity of both conditions.^[4]^

The role of pathogenic bacteria in the evolution of PI cannot be understated. Bacterial strains not only initiate the infectious process but also influence the subsequent inflammatory response and recovery trajectory of patients.^[4]^ In light of this, the present study aims to integrate the analysis of bacterial strain characteristics into the broader evaluation of prognostic factors in patients with CI complicated by PI. A comprehensive understanding of the distribution and antibiotic sensitivity profiles of these pathogens may offer valuable insights into optimizing therapeutic interventions, ultimately aiming to mitigate the high mortality associated with PI in the context of CI.

## 2. Materials and methods

### 2.1. Study design

This retrospective study included 175 patients with pneumonia following CI who were diagnosed and treated at the First People’s Hospital Affiliated to Anhui University of Technology between October 2023 and October 2024. Outcomes were assessed at 90 days after discharge using the modified Rankin Scale (mRS); a favorable outcome was defined as mRS 0 to 3 and an unfavorable outcome as mRS 4 to 6. The study population was stratified into 2 groups based on clinical outcomes: a group with good prognosis and a group with poor prognosis. Comparative analysis revealed no statistically significant differences in baseline characteristics, including gender and age, between the 2 groups. All cases of CI were confirmed using head magnetic resonance imaging or computed tomography, and the diagnoses adhered to the criteria outlined in the 2020 Guidelines for the Early Management of Patients with Acute Ischemic Stroke.^[5]^ This study was conducted in strict accordance with the ethical principles outlined in the Declaration of Helsinki. This research was approved by the Ethics Committee of The First Affiliated Hospital of Anhui University of Science and Technology. Written informed consent was obtained from all participants prior to their enrollment.

### 2.2. Inclusion and exclusion criteria

*Inclusion and exclusion criteria*: patients diagnosed with CI by head magnetic resonance imaging or computed tomography in accordance with the 2020 Guidelines for the Early Management of Patients with Acute Ischemic Stroke. Patients who developed PI subsequent to the onset of CI, as confirmed by imaging examinations showing infiltrative lesions in the chest. Patients exhibiting at least 2 of the following clinical manifestations: purulent airway secretions; fever with a body temperature exceeding 38 °C; abnormal peripheral blood leukocyte count (either >10 × 10^9^/L or <4 × 10^9^/L). *Exclusion criteria*: patients who presented with PI at the time of admission or immediately prior to admission. Cases with incomplete or insufficient clinical data for definitive diagnosis and analysis. Patients with other pulmonary conditions that may mimic the clinical and radiographic features of pneumonia, such as tuberculosis, neoplastic lesions, pulmonary edema, atelectasis, or pulmonary embolism. Patients with significant mental or speech disorders that could impede accurate clinical assessment or communication.

### 2.3. Data collection

Baseline patient information was collected, encompassing personal characteristics such as age, gender, and history of CI; vascular risk factors including hypertension, diabetes mellitus, atrial fibrillation, coronary artery disease, and smoking or alcohol consumption; therapeutic interventions following admission, such as endotracheal intubation, ventilator use, ICU admission, and the presence of invasive procedures; as well as laboratory parameters including the neutrophil-to-lymphocyte ratio (NLR), C-reactive protein/albumin ratio (CAR), procalcitonin, and fasting blood glucose levels.

### 2.4. Pathogenic bacteria culture and drug sensitivity test

Sputum specimens were obtained from patients diagnosed with PI. For those able to expectorate, patients were instructed to cough deeply into a sterile sputum container after rinsing their mouths with water in the early morning. For patients unable to produce sputum spontaneously, sterile suction tubes were employed to collect samples. In mechanically ventilated patients, alveolar lavage fluid was acquired at the bedside using a fiberoptic bronchoscope.

To ensure specimen quality, all sputum samples underwent microscopic screening under low-power magnification (×100) before culture. Specimens with <10 squamous epithelial cells and >25 polymorphonuclear leukocytes per low-power field were deemed qualified for culture. Unqualified specimens were rejected, and new samples were collected.

Qualified specimens were subjected to smear staining and microscopic examination, followed by inoculation onto blood agar and chocolate agar plates, which were incubated at 35 to 37 °C for 3 to 5 days under appropriate atmospheric conditions. Pathogen identification was performed using the CYHG EXS 1000, a fully automated microbial mass spectrometry detection system based on MALDI-TOF MS.

Antimicrobial susceptibility testing was carried out using the Kirby–Bauer disk diffusion method and/or automated systems, and the results were interpreted according to the Clinical and Laboratory Standards Institute guidelines (2023 edition). For certain antimicrobial agents or when Clinical and Laboratory Standards Institute breakpoints were unavailable, European Committee on Antimicrobial Susceptibility Testing standards were applied. Multidrug resistance was defined according to the criteria established by the European Centre for Disease Prevention and Control and the U.S. Centers for Disease Control and Prevention.

### 2.5. Statistical analysis

Data analysis was performed using SPSS Statistics version 27.0 (IBM Corp., Armonk) and R version 4.4.1 (R Foundation for Statistical Computing, Vienna, Austria). Categorical variables were expressed as percentages and compared using the Chi-square test. Continuous variables were first tested for normality. Those that were normally distributed were reported as mean ± standard deviation ($\bar{x}$ ± s) and compared using the independent samples *t* test. Variables that did not conform to a normal distribution were expressed as medians with interquartile ranges and analyzed using the Mann–Whitney *U* test. A 2-tailed *P*-value of <.05 was considered statistically significant.

The sample size was determined a priori for multivariable logistic regression, assuming an unfavorable outcome rate of ~33%, an odds ratio of 2.0 for a key predictor, α = 0.05, and 80% power. This yielded a minimum of ~164 participants; allowing for 5% to 7% attrition, the target was set at 175. With 5 prespecified predictors and >50 events, the model met the ≥10 events-per-variable criterion for stability.

In developing the prognostic model, variables with *P* < .05 in the univariate analysis were included in the multivariate logistic regression. Before inclusion, potential multicollinearity among variables was assessed using the variance inflation factor, and variables with variance inflation factor >5 were excluded to reduce collinearity effects. Variable selection in the multivariate model was performed using a stepwise regression method (both forward and backward selection based on the likelihood ratio test) to identify the most parsimonious model.

Missing data were handled using a complete case analysis approach; only cases with complete data for the variables of interest were included in the regression models. A nomogram was then constructed to visualize the model. The model’s discriminative ability and predictive performance were evaluated by the receiver operating characteristic curve and the area under the curve (AUC). Internal validation was performed using the Bootstrap method with 1000 resamples.^[6]^ The goodness-of-fit of the model was assessed using the Hosmer–Lemeshow test, and calibration curves were plotted to evaluate its accuracy.^[7]^

## 3. Results

### 3.1. Distribution of pathogenic bacteria in study participants

A total of 169 pathogenic isolates were obtained from sputum specimens of 175 patients, with multispecies infections detected in 28 patients. Among the isolates, 107 (63.31%) were gram-negative bacteria, 41 (24.26%) were gram-positive bacteria, and 21 (12.43%) were fungi. The predominant gram-negative bacteria were *Klebsiella pneumoniae* (20.12%), *Pseudomonas aeruginosa* (14.20%), and *Acinetobacter baumannii* (13.02%). Within the gram-positive group, *Staphylococcus aureus* was most frequently identified (9.46%), followed by *Streptococcus pneumoniae* (5.33%) and *Staphylococcus epidermidis* (4.14%). *Candida albicans* was the main fungal pathogen, accounting for 10.06% of the isolates (Table 1).

**Table 1:** The distribution and proportion of pathogenic bacteria.

Pathogenic bacteria	Case	Constituent ratio (%)
Gram-negative bacterium	107	63.31
*Klebsiella pneumoniae*	34	20.12
*Pseudomonas aeruginosa*	24	14.2
*Acinetobacter baumannii*	22	13.02
*Escherichia coli*	7	4.14
*Stenotrophomonas maltophilia*	5	2.96
Others	15	8.87
Gram-positive Bacterium	41	24.26
*Staphylococcus aureus*	16	9.46
*Streptococcus pneumoniae*	9	5.33
*Staphylococcus epidermidis*	7	4.14
Others	9	5.33
Fungus	21	12.43
*Candida albicans*	17	10.06
Others	4	2.37
*Total*	169	100

### 3.2. Drug resistance in gram-negative bacteria

*K pneumoniae* exhibited a high resistance rate of 67.6% to ampicillin and demonstrated elevated resistance to carbapenems, cephalosporins, and aminoglycosides. *P aeruginosa* showed resistance rates exceeding 60% to ampicillin/sulbactam, cotrimoxazole, and tigecycline. *A baumannii*, meanwhile, presented with a high level of resistance across all tested antimicrobial agents (Table 2).

**Table 2:** Resistance rate of major gram-negative bacilli to commonly used drugs (%).

Name of drug	*Klebsiella pneumonia* (n = 34)		*Pseudomonas aeruginosa* (n = 24)		*Acinetobacter baumannii* (n = 22)	
	Case	Drug-resistance rates (%)	Case	Drug-resistance rates (%)	Case	Drug-resistance rates (%)
Meropenem	7	20.6	5	20.8	13	59.1
Imipenem	7	20.6	6	25	13	59.1
Levofloxacin	12	35.3	7	29.2	12	54.5
Piperacillin/Tazobactam	11	32.4	6	25	12	54.5
Piperacillin	3	8.8	6	25	0	0
Ampicillin	23	67.6	0	0	9	40.9
Piperacillin/Sulbactam	14	41.2	15	62.5	10	45.5
Gentamicin	9	26.5	3	12.5	14	63.6
Amikacin	9	26.5	2	8.3	13	59.1
Ceftazidime	10	29.4	5	20.8	13	59.1
Cefotaxime	12	35.3	0	0	13	59.1
Cefepime	10	29.4	3	12.5	12	54.5
Cotrimoxazole	11	32.4	17	70.8	13	59.1
Tigecycline	9	26.5	15	66.7	0	0
Chloramphenicol	10	29.4	14	58.3	10	45.5

### 3.3. Prognostic factors of CI patients with PI

Among the 175 patients, 116 were categorized in the good prognosis group and 59 in the poor prognosis group. There was no statistically significant difference in age (*t* = 1.345, *P* = .180) or gender (*χ*² = 1.828, *P* = .176) between the 2 groups. Statistically significant differences were observed regarding ICU admission, the presence of atrial fibrillation, impaired consciousness, the occurrence of invasive procedures, as well as laboratory parameters including the NLR and CAR, and the incidence of multispecies infections (*P* < .05). In contrast, no significant differences were found in terms of history of infarction, hypertension, diabetes mellitus, and the prevalence of gram-negative bacterial infections (*P* > .05) (Table 3).

**Table 3:** Analysis of prognostic factors in CI patients with pulmonary infections.

Factors	Poor prognosis (n = 59)	Good prognosis (n = 116)		*P*-value
Age	75.31 ± 11.20	72.93 ± 10.97	1.345	.18
Gender				
Male	33 (37.1)	77 (72.9)	1.828	.176
Female	26 (21.9)	39 (43.1)		
ICU				
No	38 (50.6)	112 (99.4)	33.002	<.001
Yes	21 (8.4)	4 (16.6)		
Infarction history				
No	36 (31.4)	57 (61.6)	2.216	.137
Yes	23 (27.6)	59 (54.4)		
Hypertension				
No	21 (18.9)	35 (37.1)	0.528	.467
Yes	38 (40.1)	81 (78.9)		
Diabetes mellitus				
No	39 (39.1)	77 (76.9)	0.001	.971
Yes	20 (9.9)	39 (39.1)		
Atrial fibrillation				
No	44 (48.9)	101 (96.1)	4.297	.038
Yes	15 (0.1)	15 (9.9)		
Intrusive operation				
No	18 (37.1)	92 (72.9)	38.895	<.001
Yes	41 (21.9)	24 (43.1)		
Impaired consciousness				
No	12 (38.8)	103 (76.2)	81.339	<.001
Yes	47 (20.2)	13 (39.8)		
NLR	11.26 (6.98–18.82)	4.41 (2.36–6.93)	-6.991	<.001
CAR	2.87 (1–4.99)	0.37 (0.06–1.29)	-6.105	<.001
G^−^ infections				
No	25 (31.0)	67 (61.0)	3.713	.054
Yes	34 (28.0)	49 (55.0)		
Multiple infections				
No	45 (49.6)	102 (97.4)	3.956	.047
Yes	14 (9.6)	14 (18.6)		

CI = cerebral infarction.

### 3.4. Prognostic logistic regression analysis of CI patients with pulmonary infection

A logistic regression model was developed to investigate the factors influencing the prognosis of PI in patients with CI. Prognosis served as the dependent variable, while the independent variables included atrial fibrillation (coded as 0 for absence and 1 for presence), multiple infections (coded as 0 for absence and 1 for presence), invasive procedures (coded as 0 for absence and 1 for presence), as well as the NLR and the CAR. The results of this analysis are detailed in Table 4.

**Table 4:** Prognostic logistic regression analysis of CI patients with pulmonary infections.

Factors	B	SE	Wald	*P*	OR	95% CI
Atrial fibrillation	1.6	0.554	8.344	.004	4.953	1.673–14.666
Multiple infections	1.385	0.558	6.16	.013	3.993	1.338–11.918
Intrusive operation	1.836	0.445	17.038	<.001	6.273	2.623–15.000
NLR	0.06	0.026	5.281	.022	1.062	1.009–1.118
CAR	0.464	0.126	13.488	<.001	1.591	1.242–2.038

CI = cerebral infarction, CAR = C-reactive protein/albumin ratio, NLR = neutrophil-to-lymphocyte ratio.

### 3.5. Construction of disease risk-prognostic models

**Figure 1.**
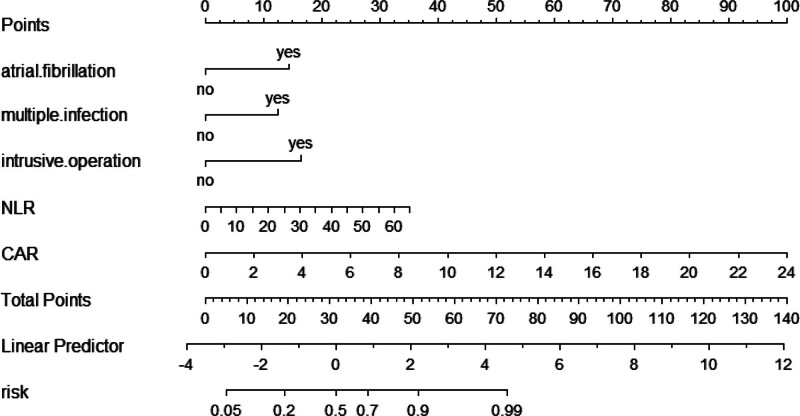
The nomogram for risk-prognostic model.

**Figure 2.**
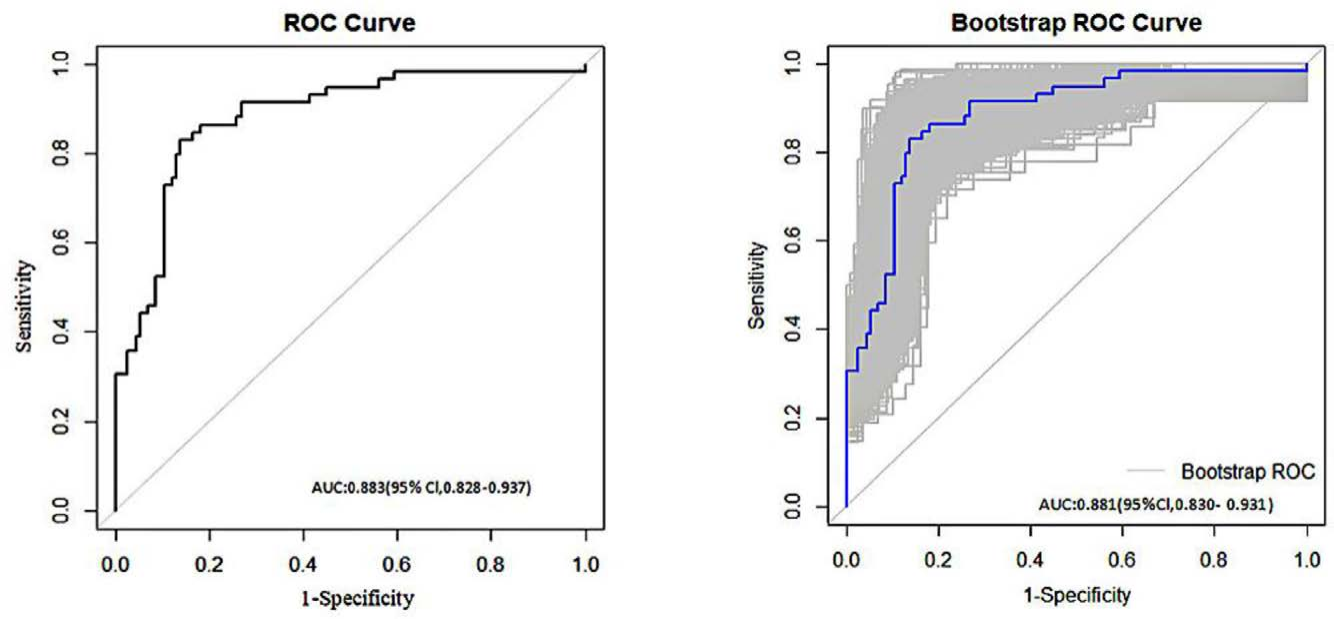
ROC curves for risk-prognostic model. ROC = receiver operating characteristic.

**Figure 3.**
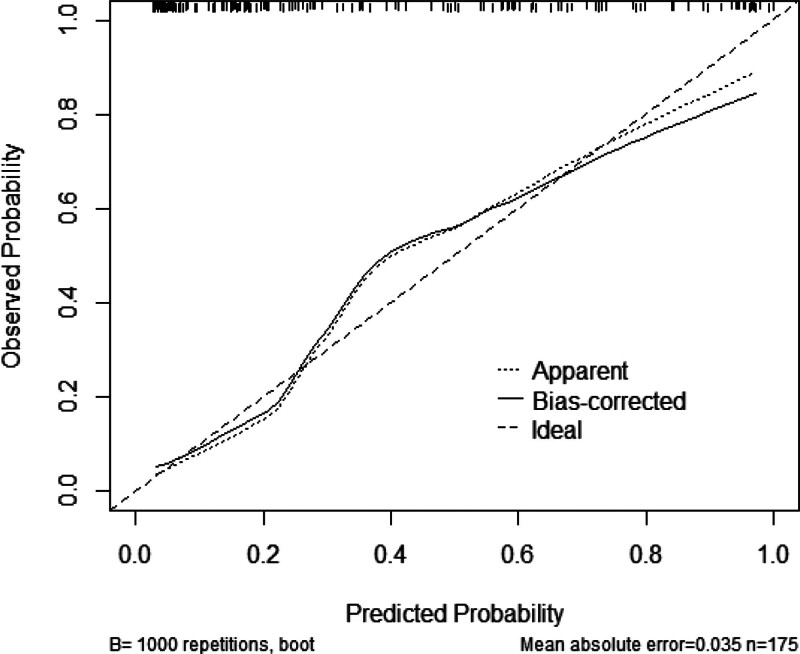
Calibration curves for risk-prognostic model.

A logistic regression model was constructed based on the analysis results, with the following equation: Logit(*P*) = 1.600 × atrial fibrillation + 1.385 × multiple infections + 1.836 × invasive procedures + 0.060 × NLR + 0.464 × CAR − 3.507. A nomogram was then generated, in which the vertical axis represents the variables and the horizontal axis corresponds to the patient-specific values. Individual scores were calculated, allowing for the estimation of the risk of PI in patients with CI (Fig. 1). Receiver operating characteristic curve analysis demonstrated that the predictive model achieved an AUC of 0.883 (95% CI: 0.828–0.937) with a Youden index of 0.693, corresponding to a sensitivity of 83.1% and a specificity of 86.2%. Internal validation via the Bootstrap method (1000 resamples) yielded an AUC of 0.881 (95% CI: 0.830–0.931) (Fig. 2). The Hosmer–Lemeshow goodness-of-fit test resulted in χ² = 11.335 (*P* = .183), indicating an acceptable model fit. Consequently, a calibration curve was plotted (Fig. 3), and the proximity of the curve’s slope to 1 suggests that the model’s predictions are in good agreement with the observed outcomes.

## 4. Discussion

It has been reported that there is a close link between infections and central nervous system diseases, including CI, Parkinson disease, and Alzheimer disease,^[8]^ which can cause damage to the blood–brain barrier through various inflammatory cytokines, as well as triggering immunosuppressive responses and exacerbating brain tissue damage, thereby dramatically exacerbating mortality rates in patients with CI, pushing up healthcare costs, and profoundly affecting prognosis. Therefore, this study aims to assess the clinical prognosis of patients with CI complicated by PI. It is worth noting that the infected pathogenic bacteria are prone to antibiotic resistance,^[9]^ which will not only impede the recovery process of patients, but also lead to an increase in the mortality rate, making the treatment particularly difficult, therefore, analyzing the characteristics of the pathogenic bacteria in patients and incorporating their characteristics into the model has important clinical and research value.

Previous studies have shown that lung infections in patients with acute CI are prevalent with gram-negative bacteria, which accounted for 75.6% of all pathogens,^[10]^ with a high detection rate of *P aeruginosa*, *A baumannii*/Haemolyticus immobilizers, *K pneumoniae*, and *Escherichia coli*. In this paper gram-negative bacteria accounted for 63.31% (107/169), including *K pneumoniae* 20.12% (34/107), *P aeruginosa* 14.20% (24/107), and *A baumannii* 13.02% (22/107), which is similar to the results of other studies.^[4]^ The reason may be that the use of ventilators is easy to lead to cross-infection in patients, causing an increase in gram-negative bacteria,^[11]^ In addition, disorders in the intestinal flora after CI lead to an increase in the permeability of intestinal epithelial tissues, which in turn also triggers the migration of intestinal flora to the lung tissues resulting in PIs,^[12]^ which contributes to the increase in the proportion of gram-negative bacteria in the flora. The pathogens are highly susceptible to drug resistance, especially *A baumannii*.^[13]^ And the mechanism of resistance is related to the fact that *A baumannii* is prone to produce β-lactamase during growth,^[14]^ which is able to hydrolyze the β-lactam ring of β-lactam antibiotics, causing them to lose their antimicrobial activity, which leads to the development of drug resistance. In this study, the drug resistance rates of *A baumannii* were higher; *K pneumoniae* was highly resistant to ampicillin at67.6%; and *P aeruginosa* was resistant to ampicillin/sulbactam, and cotrimoxazole at >60%. In conclusion, the multidrug resistance situation of PI pathogens is becoming increasingly severe, and it is very important to select effective antibiotics early so as to avoid the development of multidrug resistance.

In this study, we successfully constructed a model for predicting the prognostic situation of PIs and plotted nomogram for visual analysis, which can help physicians to identify high-risk patients at an early stage and thus significantly improve the overall prognosis of patients. The model, which includes 5 variables including atrial fibrillation, multiple infections, intrusive operation, NLR, and CAR, has good discriminatory ability and has potential application in clinical settings.

Atrial fibrillation (AF) is one of the most common cardiac arrhythmias that can cause cardiogenic CI, which accounts for about one-fifth of the incidence of CI.^[15]^ Studies have shown that the occurrence and prognosis of CI is the result of a combination of factors, including AF.^[16]^ Patients with atrial fibrillation tend to have irregular heart rhythms, and the episodes can increase the body’s fibrinogen expression and decrease lymphocytes, which increases the probability of infection in the body. In addition the heart pumping function decreases, which in turn affects the body’s immunity and resistance, making it more difficult to treat. Han suggested that patients with AF have a higher risk of early infection,^[17]^ disability and death than patients without AF. Therefore, we hypothesized that AF may be associated with the prognosis of PI. Multiple bacterial infections are a state of infection caused by the simultaneous or sequential invasion of 2 or more different types of pathogens into the body. Some patients with PIs have a disturbed immune system, and the occurrence of multiple infections results in a synergistic effect through bacterial interactions, leading to a more severe inflammatory response and damage to the immune system, and an increase in patient mortality. In addition, this study found that invasive operations are also associated with poor patient prognosis, some patients need mechanical ventilation to maintain life during diagnosis and treatment, and various types of luminal intubation can cause the destruction of the skin mucosa, the body’s first defense barrier, which increases the chance of invasion by pathogenic bacteria, resulting in a poorer prognosis. Wu demonstrated that prolonged tracheotomy is an important multidrug-resistant bacterial infection pneumonia after stroke risk factors.^[18]^ Neutrophil and lymphocyte count ratio, as an inflammatory marker, has been gaining attention in recent years. NLR can reflect the balance between lymphocytes (inflammation regulators) and neutrophils (inflammation activators),^[19]^ so it can reflect the inflammatory response of the organism and the status of infection more sensitively. Moreover, NLR can be obtained quickly and easily from different blood cell counts, which can help timely intervention in high-risk patients. A study showed that NLR has high sensitivity for the recognition of stroke-associated pneumonia (SAP).^[20]^ Nam et al suggested that SAP patients with high NLR had poorer clinical outcomes during hospitalization and after discharge,^[21]^ which is in agreement with the results of our study, so NLR can also be used for prognostic assessment. C-reactive protein and albumin are markers synthesized by the liver. The process of lung infection triggers a series of cascade reactions mediated by inflammatory cells, and there is a sharp increase in C-reactive protein in the organism, while ALB is consumed in large quantities, so CAR levels increase significantly and amplify inflammatory signals, better reflecting the state of inflammation, and thus the prognosis.^[22]^ Stroke patients with higher CAR levels are more likely to be hospitalized while develop SAP, have poorer short-term clinical outcomes, and have higher rates of mortality and severe disability at 3 months.^[23]^ In our study, it was shown that patients with high CAR had a poorer prognosis. Therefore, it can be assumed that there is a link between CAR and prognosis, and dynamic monitoring of CAR can help to intervene in high-risk patients in a timely manner and improve patient care.

The proposed prognostic nomogram integrates multiple clinical and laboratory parameters, enabling clinicians to identify high-risk patients early, optimize antimicrobial regimens, and strengthen monitoring and supportive measures. Its visual format allows for quick risk estimation at the bedside, making it feasible for use in various healthcare settings, including tertiary hospitals, rehabilitation wards, and primary care facilities. This may help standardize the assessment of CI patients with PI and facilitate individualized treatment strategies.

This study has several limitations. First, its retrospective design may introduce selection bias and precludes establishing causal relationships. Second, the single-center nature of the study may limit the generalizability of our findings to other populations or healthcare systems. In addition, although our sample size was adequate for model construction, it may not fully represent the heterogeneity of pathogen distribution and clinical characteristics seen in different regions or institutions. Furthermore, unmeasured confounding factors may still exist.

## 5. Conclusions

In summary, this study helped us to identify a history of atrial fibrillation, multiple infections, intrusive operation, elevated NLR, and elevated CAR as independent risk factors for the prognosis of CI with PI and may serve as relevant markers for treatment. The nomogram confirmed this finding and was subjected to Bootstrap internal validation, providing new ideas for clinical studies. In addition, to our knowledge, few studies have included pathogenic bacteria characteristics in the study of patient prognosis. However, the present study is a retrospective analysis with a small sample size and all cases were from our hospital, which has some limitations and shortcomings. In view of this, future research should focus on prospective validation in 3 to 5 stroke centers with diverse patient populations and varied antibiotic stewardship protocols, enabling assessment of the model’s robustness across different clinical settings and improving its generalizability.

## Author contributions

**Conceptualization:** Suxia Hu, Zejin Wang, Lei Zhu, Yifei Zhu.

**Data curation:** Suxia Hu, Zejin Wang, Lei Zhu, Yifei Zhu.

**Formal analysis:** Suxia Hu, Zejin Wang, Yifei Zhu.

**Investigation:** Suxia Hu, Zejin Wang, Yifei Zhu.

**Methodology:** Suxia Hu, Zejin Wang, Yifei Zhu.

**Supervision:** Suxia Hu, Lei Zhu, Yifei Zhu.

**Validation:** Lei Zhu.

**Visualization:** Suxia Hu, Lei Zhu.

**Writing – original draft:** Suxia Hu, Zejin Wang.

**Writing – review & editing:** Suxia Hu, Zejin Wang.
